# The Influence of Physical Therapy Guideline Adherence on Healthcare Utilization and Costs among Patients with Low Back Pain: A Systematic Review of the Literature

**DOI:** 10.1371/journal.pone.0156799

**Published:** 2016-06-10

**Authors:** William J. Hanney, Michael Masaracchio, Xinliang Liu, Morey J. Kolber

**Affiliations:** 1 Department of Health Professions, University of Central Florida, Orlando, Florida, United States of America; 2 Department of Physical Therapy, Long Island University, Brooklyn, New York, United States of America; 3 Department of Health Management and Informatics, University of Central Florida, Orlando, Florida, United States of America; 4 Department of Physical Therapy, Nova Southeastern University, Fort Lauderdale, Florida, United States of America; University of Exeter, UNITED KINGDOM

## Abstract

**Background:**

Low back pain (LBP) is common and associated healthcare costs are significant. While clinical practice guidelines have been established in an attempt to reduce costs and healthcare utilization, it is unclear if adherence to physical therapy guidelines for those with LBP is efficacious. Therefore, the purpose of this study was to assess current evidence and evaluate the impact of physical therapy guideline adherence on subsequent healthcare costs and utilization for patients with LBP.

**Methods:**

An electronic search was conducted in PubMed, CINAHL (EBSCO Host), AMED (Ovid), and PEDro. Studies included in this review were published in peer reviewed journals and the primary mode of treatment was administered by a physical therapist. Also, the definition of adherence was clearly defined based on claims data and at least one measure of cost or utilization reported. Quality assessment was evaluated via a modified Downs and Black checklist. Due to the conceptual heterogeneity in variable measurements, data were qualitatively synthesized and stratified by reported utilization and cost measures.

**Results:**

A total of 256 results were identified and after omitting duplicates, 4 articles were retained, which were all retrospective in nature. Quality scores ranged between 19 and 21 points out of a possible 26 on the modified Downs and Black checklist. All identified studies used the same definition of guideline adherence, which focused on billing active codes and minimizing use of passive codes. The results demonstrated trends that, with a few exceptions, suggested those patients with LBP that were treated with an adherent guideline program demonstrated decreased healthcare utilization and an overall healthcare savings.

**Conclusion:**

Preliminary evidence suggests that adherence to established clinical practice guidelines may assist with decreasing healthcare utilization and costs. Additional research based on prospective randomized controlled trials are needed to provide high quality evidence regarding the impact of guideline adherence among patients with LBP.

## Introduction

Evidence suggests that up to 84% of the general population will report at least one episode of low back pain (LBP) at some point in their lifetime [1, 2]. LBP is one of the most common reasons for an individual to consult with their physician [3], and current research has reported that direct and indirect healthcare costs associated with the treatment of LBP account for between 85 and 238 billion dollars each year [3, 4]. For this reason, the Institute of Medicine has identified LBP as one of the top 15 priority conditions in the United States [5]. The prevalence and cost of LBP has led to debate regarding how to best manage this condition. The majority of LBP episodes will resolve within 2 to 4 weeks. However, recurrent episodes over a given year have been reported in one study to be as high as 25% [6]. Considering the high prevalence rates and incidence of recurrent episodes, LBP is primed to be an epidemic in the 21^st^ century. In order to deter the economic and clinical influences of LBP, proposed standardization has been a topic of considerable interest.

When analyzing clinical practice patterns, it seems that the treatment of LBP is not consistent with the current general recommendations [7, 8]. In a recent survey-based study [9], only 30% of musculoskeletal physical therapists adhered to evidence based guidelines and this rate drops to 15% if the clinician is not a credentialed musculoskeletal specialist. Of particular concern is the excessive cost of care accumulated early in the treatment process, which is potentially attributed to this inconsistency in adhering to evidence based practice [6, 7]. As a result, clinical practice guidelines (CPG) have been developed as a manner by which to standardize care for LBP. These guidelines are often developed by expert consensus panels and generally assist clinicians in identifying treatment options and specific recommendations for this population utilizing the best available evidence. Grimshaw et al[10] suggested CPGs may decrease the utilization of ineffective treatment options, promote evidence-based practice, and improve patient outcomes.

While many professionals are involved in the management of musculoskeletal disorders, physical therapists are a significant provider of conservative care for patients with LBP [11]. Some guidelines have recommended delaying physical therapy referrals for 2 to 4 weeks in order to allow an opportunity for spontaneous recovery [12–14]. However, other studies have identified an overall cost savings with early referral to physical therapy [15, 16]. Childs et al[17] found those patients undergoing physical therapy for LBP that participated in a treatment program reflective of current guidelines demonstrated less overall healthcare utilization and costs. Conversely, other studies suggest there are little cost savings in referring to physical therapy early. Fritz et al [18] found while there was not an increase in costs associated with early physical therapy, there was little cost benefit. This conflict, in part, has contributed greater interest in how adherence to CPGs for physical therapy treatment of LBP may influence healthcare utilization and costs.

Guidelines for LBP were first published by the Quebec Task Force in 1987, and the authors concluded that there is a lack of high quality evidence to assist clinicians in decision making [19]. Since then, several treatment guidelines have been developed to assist in the management of LBP which support an active approach to rehabilitation while minimizing bed rest and passive modalities. [20–26]. Guidelines have been adopted by several organizations for physical therapy and generally recommend an active treatment approach with the primary goal of educating patients to regain control over the condition [27–29]. Also, while the majority of physical therapists strongly agree with general recommendations for treatment of LBP [30–32], it seems their use of CPGs varies widely [33]. While considerable attention has focused on guideline adherence, few studies examined the impact of guideline adherence on healthcare utilization and costs among patients with LBP. To our knowledge no systematic review has been conducted on this topic. Therefore, the purpose of this study was to assess the current body of evidence and evaluate the impact of physical therapy guideline adherence on healthcare utilization and costs in patients with LBP.

## Methods

### Data Sources and Searches

An electronic search was conducted in PubMed, CINAHL (EBSCO Host), AMED (Ovid), and PEDro May 3^rd^, 2016. Key words were used independently and in combination including, *guideline adherence*, *low back pain*, *cost*, *utilization*, *physical therapy* and *physiotherapy*. Specific search strategies are outlined in Table 1.

**Table 1:** Database Search Strategies.

Database	Search Strategy	Results
PubMed	("physical therapy" OR "physiotherapy") OR "low back pain" AND ("cost" AND "utilization")	140
CINAHL (EBSCO Host)	physical therapy OR physiotherapy (select a field (optional) OR low back pain (select a field (optional) AND (cost OR utilization (select a field (optional))	54
AMED (Ovid)	("physical therapy" OR "physiotherapy") OR "low back pain" AND ("Cost" AND "Utilization")	48
PEDro	Simple search: physical therapy, low back pain, Cost, Utilization	14

### Study Selections

Inclusion criteria for studies included in this review were: (1) published in a peer reviewed journal, (2) published in the English language, (3) primary mode of treatment administered by a physical therapist, (4) definition of adherence clearly defined based on claims data and (5) at least one measure of cost or utilization reported and (6) the study design was retrospective, prospective cohort, or a randomized controlled trial. Narrative reviews, commentaries, case studies, and case series were excluded from the review. Initial inclusion was considered if keywords were found in the title or abstract and the article met the inclusion criteria.

### Data Extraction

Two reviewers (WJH & MM) examined all titles and abstracts independently to determine initial study eligibility and then separately re-evaluated full text articles for specific inclusion criterion. A third reviewer (XL) determined final eligibility when a discrepancy existed between the initial reviewers. Data regarding the primary characteristics, which included cost and healthcare utilization, were extracted from the identified articles. Primary variables evaluated for healthcare utilization included number of physical therapy visits, duration of physical therapy care, prescription medication use, opioid medication use, additional physician office visits, emergency department care, advanced imaging, diagnostic procedures, surgical procedures, and injection procedures. Variables evaluated for healthcare cost included physical therapy, prescription medications, physician office visits, imaging procedures, surgical/injection procedures, inpatient non-surgical procedures, inpatient costs, total LBP costs, other LBP related costs, non-LBP healthcare costs, and subsequent healthcare costs.

### Quality Assessment

Methodological quality of each study was independently evaluated using the Downs and Black checklist [34]. The Downs and Black checklist is a scale which can be used to evaluate study quality for those which are non-randomized in nature. The Downs and Black checklist includes four different categories of assessment that include reporting, external validity, internal validity/bias, and internal validity/confounding. The Downs and Black has demonstrated good inter-rater reliability (r = 0.75) and test-retest reliability (r = 0.88).[34] Also, a high internal consistency has been reported (KR-20 = 0.98). Each study was further evaluated for significant results (P< 0.05) in the outcome categories of cost or healthcare utilization.

The version of the Downs and Black scale used was modified based on previously published reports S1 Table [35]. Item 17; “In trials and cohort studies, do the analyses adjust for different lengths of follow-up of patients, or, in case-control studies, is the time period between the intervention and outcome the same for cases and controls?” lacked assessment of quality in the identified studies because of the largely cross sectional nature of the design. Item 27; “Did the study have sufficient power to detect a clinically important effect where the probability value for a difference being due to chance is less than 5%” was also omitted due to reports suggesting post hoc power analysis should not influence significance. Specifically, it was suggested that differences between groups are either significant or not upon termination of the study independently of assumptions made *a priori* [36].

Each of the identified studies was scored using the modified Black and Downs Scale by two independent reviewers (WJH and MM). The results of these scoring tests were blinded for each of the evaluators and a third author (XL) evaluated inconsistent scores. Finally, because identified studies were based on claims data and were not randomized controlled trials, risk of bias was evaluated using the study limitations of observational studies [34].

### Data Synthesis and Analysis

After evaluating each article for quality based on the modified Downs and Black scale the data were synthesized by the primary author (WJH). Data was then stratified by outcome measure for analysis. The Modified Downs and Black scale was scored out of a possible 26 points and a modified Downs & Black scale score of 20–26 was considered excellent, 15–19 good, 10–14 fair and </ = 9 poor. The results were evaluated for support of guideline adherence and guideline non-adherence. A variable was considered favoring guidelines adherence if ≥ 50% of studies which were of good to excellent quality demonstrated statistically significantly differences. Guideline non-adherence was supported if ≥ 50% of studies which were of good to excellent quality demonstrated statistically significantly differences. Finally, no difference was supported if there was no statistically significant difference between guideline adherence and guideline non-adherence. The authors considered utilizing a meta-analysis however, in closer evaluation of the reported data it was noted that the time frame in which the data was considered differs between studies. For example, studies reported cost and utilization measures over a 12, 18, or 24 month period of time. While the variables and methodology may seem similar the time differences create variables which are unique. Therefore, a meta-analysis would not be suitable for the current study (Table 2).

**Table 2:** Data Synthesis.

	Outcome Measure	Summary	Favors
**Healthcare Utilization**	# of PT visits	Three studies [17, 37, 38] with good to excellent quality reporting on # of PT visits demonstrated significant differences favoring guideline adherence.	GA
	Duration of PT Care	Two studies [37, 38] with good to excellent quality reported on the Duration of PT care with significant difference favoring guideline adherence.	GA
	Prescription Medication	One study [38] with good quality reported on prescription medication utilization with significant differences favoring guideline adherence.	GA
	Opioid Medication	Three studies [15, 17, 38] with good to excellent quality reported on utilization of opioid medication with no significant difference between in guideline adherence and non-adherence	ND
	Physician Office Visit	Two studies [15, 38] with good to excellent quality reported on physician office visit with no significant difference between guideline adherence and non-adherence	ND
	ER Care	One study [38] with good quality reported on ER care with no significant difference between guideline adherence and non-adherence	ND
	Advance Imaging	Two studies [17, 38] with good to excellent quality reported decreased utilization of advanced imaging. One study [15] showed no difference	GA
	Diagnostic Procedures	One study [38] with good quality reported on diagnostic procedures with significant differences favoring guideline adherence.	GA
	Surgical Procedures	Two studies [15, 17] with good to excellent quality reported decreased utilization of advanced imaging. One study [38] showed no difference.	GA
	Injection Procedures	Three studies [15, 17, 38] with good to excellent quality reporting on # of PT visits demonstrated significant differences favoring guideline adherence.	GA
**Healthcare Costs**	Costs of PT	Two studies [37, 38] with good to excellent quality reported decreased Costs of PT favoring guideline adherence.	GA
	Prescription Medication	One study [15] with excellent quality reported a non-significant decrease in costs and 1 additional study [17] of good quality reported a significant reduction in cost favoring guideline adherence.	GA
	Physician Office Visit	One study [15] with excellent quality reported a significant decrease in costs associated with physician office visits favoring guideline adherence.	GA
	Imaging Procedures	One study [15] with excellent quality reported non-significant differences associated with imaging procedures favoring guideline adherence.	ND
	Surgical/Injection Procedures	One study [15] with excellent quality reported non-significant differences associated with surgical/injection procedures favoring guideline adherence.	ND
	Inpatient Nonsurgical Procedures	One study [15] with excellent quality reported non-significant differences associated with inpatient nonsurgical procedures favoring guideline adherence.	ND
	Inpatient Costs	One study [17] with good quality reported non-significant differences associated with inpatient costs favoring guideline non-adherence.	ND
	Total LBP Costs	Two studies [15, 17] with good to excellent quality reported a significant decrease in total LBP costs favoring guideline adherence.	GA
	Other LBP Related Costs	One study [15] with excellent quality reported a significant decrease in other LBP related costs favoring guideline adherence.	GA
	Non-LBP Healthcare Costs	One study [15] with excellent quality reported a non-significant decrease non-LBP related costs favoring guideline adherence. Another study [17] with good quality reported a non-significant decrease in non-LBP related costs favoring guideline non-adherence.	ND
	Subsequent Healthcare Costs	One study [38] with good quality reported a non-significant decrease in subsequent healthcare costs favoring guideline adherence.	ND

GA–Guideline Adherence, GN–Guideline Non-adherence, ND–No Difference

## Results

**Fig 1:**
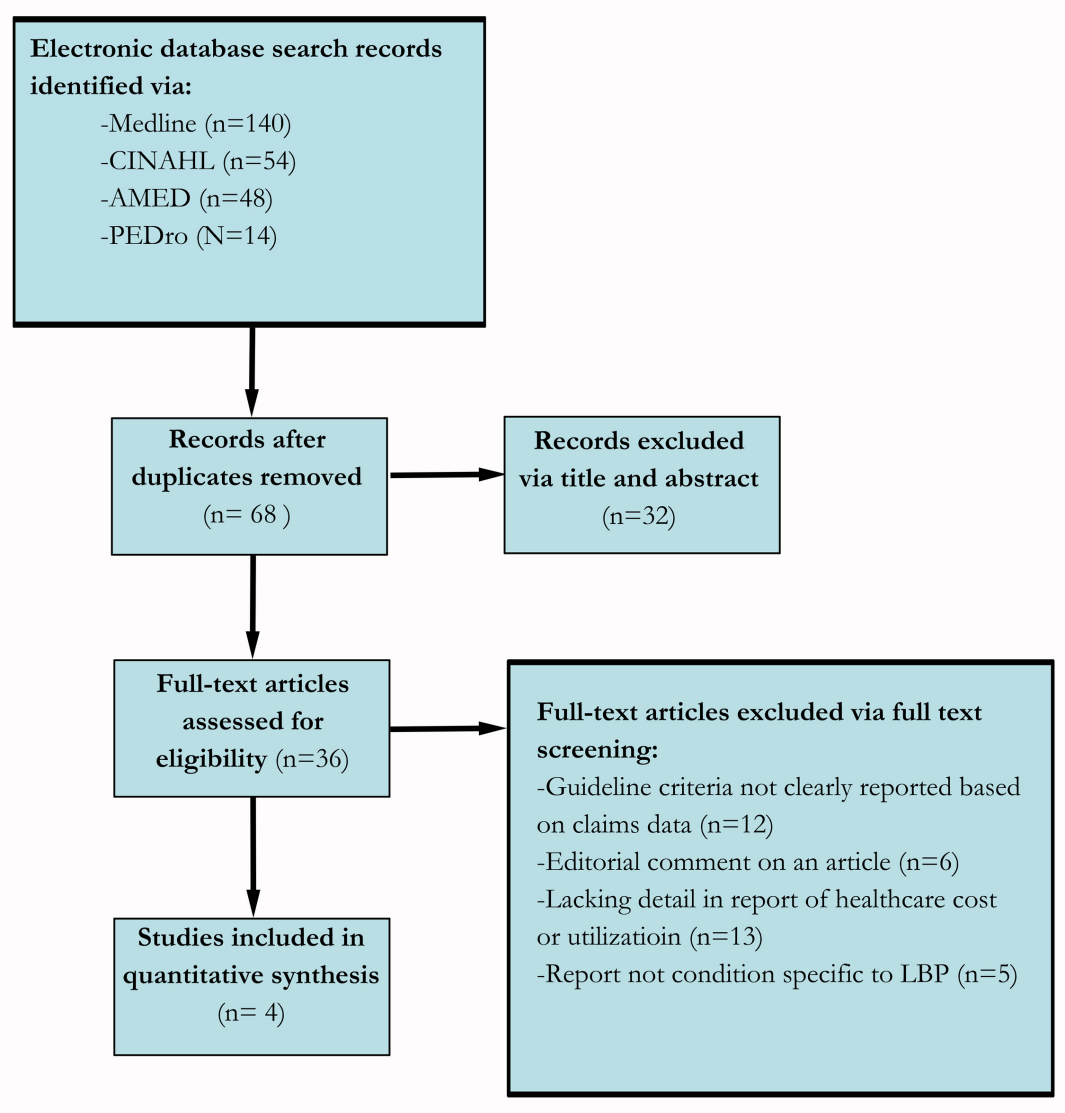
PRISMA flow diagram.

The initial search of each database results were as follows: Medline (PubMed) (140), CINAHL (EBSOCO host) (54), AMED (Ovid) (48), and PEDro (14). Thus, a total of 256 results were identified (Fig 1). After applying the inclusion criteria and omitting duplicates, 4 articles remained and were included in this review. Table 3 summarizes the primary characteristics of the studies identified. Considering a maximum total score of 26 points on the Downs and Black checklist all articles scored between 19 and 21 points (Table 4). For utilization, studies reported percentages relative to the overall sample evaluated. Costs reported represented the actual payment amounts recorded in the respective claims databases.

**Table 3:** Study Details.

Study	Data Source	Study Design	Patients Included That Utilized PT	Definition of Guideline Adherence	Patients Receiving Adherent Care	Patients Receiving Non-Adherent Care	% Adherence Reported	Healthcare Utilization Identified	Healthcare Cost Identified
Fritz et al,[37] 2007	Clinical outcomes and financial data bases maintained by the Rehabilitation Agency of Intermountain Healthcare	Retrospective review	1190	Active to passive codes ≥ 75% and each visit included at least 1 active code	481	709	40	-# of PT visits, Duration of PT episode of care	Charges for PT
Fritz et al,[38] 2008	Clinical outcomes and financial data bases maintained by the Rehabilitation Agency of Intermountain Healthcare	Retrospective review	471	Active to passive codes ≥ 75% and each visit included at least 1 active code	132	339	28	# of PT visits, Duration of PT episode of care, Prescription medications, Physician office visits, Emergency/urgent care visits, Diagnostic procedures, Surgical procedures, Injection procedures, Rehabilitation visits (Chiro & PT)	Charges for PT
Fritz et al,[15] 2012	Mercer HealthOnline	Retrospective review	1,917	Active to passive codes ≥ 75% and each visit included at least 1 active code	413	1504	21.5	Advanced imaging (MRI or CT), Additional physician visits, Lumbar spine surgery, Lumbar spinal injections, Opioid medication use	Imaging procedures, Physician visits, Surgical/Injection procedures, Inpatient nonsurgical procedures, Emergency room visits, Prescription medication, Other LBP related costs, Total LBP costs, Non-LBP healthcare costs
Childs et al,[17] 2015	Military Health System Management Analysis and Reporting Tool	Retrospective review	71,559	Active to Passive codes ≥ 75% and each visit included at least 1 active code	30,917	40,642	43	# of physical therapy visits, Advanced imaging, Lumbar spinal injections, Lumbar spine surgery, Opioid medication use	Prescription medications, Inpatient costs, Total LBP costs, Non-LBP healthcare costs

PT, physical therapy; MRI, magnetic resonance imaging; CT, computed tomography; LBP, low back pain; Chiro, chiropractic.

**Table 4:** Methodological Quality.

	Reporting										External Validity			Internal Validity (Bias)							Internal Validity (Confounding)							
Study	1	2	3	4	5	6	7	8	9	10	11	12	13	14	15	16	17	18	19	20	21	22	23	24	25	26	27	D&B Score
Fritz et al,[37] 2007	Y	Y	Y	Y	P	Y	Y	N	N	Y	Y	Y	Y	Y	Y	Y	-	Y	Y	Y	Y	Y	N	N	Y	U	-	20/26
Fritz et al,[38] 2008	Y	Y	Y	Y	P	Y	Y	N	N	N	Y	Y	Y	Y	Y	Y	-	Y	Y	Y	Y	Y	N	N	Y	U	-	19/26
Fritz et al,[15] 2012	Y	Y	Y	Y	P	Y	Y	N	N	Y	Y	Y	Y	Y	Y	Y	-	Y	Y	Y	Y	Y	N	N	Y	U	-	20/26
Childs et al,[17] 2015	Y	Y	Y	Y	P	Y	Y	N	N	N	Y	Y	Y	Y	Y	Y	-	Y	Y	Y	Y	Y	N	N	Y	U		19/26

Criteria based on modified Downs and Black checklist: Y (yes) = criterion met, N (no) criterion not met, P (partial) criterion partially met, and U (unable to determine) Criteria unable to determined.

### Adherence to Guideline Recommendations

Considerable variability existed in reported guideline adherence with non-adherence ranging from 60% to 78%. Fritz et al [37] reported of 1190 patients receiving physical therapy, 481 (40%) received care consistent with guideline recommendations, while 709 (60%) received non-adherent care. A subsequent study published by Fritz et al [38] evaluated a total of 471 subjects and found 132 (28%) received adherent care and 339 (72%) received non-adherent care. Another study was able to evaluate 1917 patients for compliance to physical therapy guidelines. They found approximately 413 (22%) received adherent care while 1504 (78%) received non-adherent care [15]. Finally, a study by Childs et al [17] reported on 71,559 individuals receiving physical therapy for LBP and found that 30,917 (43%) were adherent and 40,642 (57%) were non-adherent [17]. While there was some variability there seemed to be consistent differences in adherence regardless of the population. For example, Childs et al [17] reported on data from the Military Heath System while the other studies utilized data from civilian populations. It seems as if there may be slightly greater adherence in the military population reporting 43% while other studies reports between 22 and 40%.

### Healthcare Utilization

#### Physical therapy visits

One measure of healthcare utilization reported in three of the identified studies was number of physical therapy visits (Table 5) [17, 37, 38]. Based on the available evidence, it seems those patients participating in an adherent physical therapy treatment program were likely to utilize fewer physical therapy visits. Fritz et al [37] concluded that patients receiving adherent care received a mean of 5 visits, while those receiving non-adherent care demonstrated a mean of 6 visits. This is consistent with a subsequent study by Fritz et al [38] that reported a mean number of PT visits to be 4.6 in the adherent group, compared to a mean of 5.9 visits in the non-adherent group. Another study by Fritz et al [15] evaluated claims data to evaluate healthcare utilization and costs. This study reported the mean number of PT sessions to be 5.3 for those receiving adherent care, compared to 7.9 for those receiving non-adherent care. Finally, the study by Childs et al [17] evaluated claims data from over 71,000 patients and reported a large difference regarding the number of PT visits, which included a mean of 6.2 visits in the adherent group, compared to 15 in the non-adherent group.

**Table 5:** Measures of Healthcare Utilization.

	# PT Visits		Duration of PT Care (Days)		Prescription Medication (%)		Opioid Medication (%)		Physician Office Visit (%)		ER Care (%)		Advanced Imaging (%)		Diagnostic Procedures (%)		Surgical Procedures (%)		Injection Procedures (%)	
	A	NA	A	NA	A	NA	A	NA	A	NA	A	NA	A	NA	A	NA	A	NA	A	NA
Fritz et al[37] 2007† median (range)	5* (3–21)	6* (3–35)	22* (10–250)	26* (10–250)	NR	NR	NR	NR	NR	NR	NR	NR	NR	NR	NR	NR	NR	NR	NR	NR
Fritz et al[38] 2008‡ mean (SD)	4.6* (2.0)	5.9* (2.2)	25.4* (16.2)	29.7* (20.6)	46.2*	57.2*	30.3	38.1	19.5	25.7	1.5	3.2	8.3*	15.9*	14.4*	23.6*	3.8	3.0	9.1*	15.9*
Fritz et al[15] 2012§	NR	NR	NR	NR	NR	NR	49.6	53.2	64.4	68.8	NR	NR	38.7	43.9	NR	NR	5.1*	8.1*	12.6*	17.8*
Childs et al[17] 2015¶ mean (SD)	6.2* (7.6)	15.0* (17.2)	NR	NR	NR	NR	65.2	66.0	NR	NR	NR	NR	17.0*	22.7*	NR	NR	2.6*	3.0*	11.7*	13.8*

† # of PT visits and duration were related to an episode of care

‡ # of PT visits and duration were related to an episode of care and other measures were observed in the year after the episode

§ utilization measures were observed during the 18-month period following the index primary care visit

¶ # of PT visits and duration were related to an episode of care and unadjusted utilization measures were observed during the 2-year follow-up period

SD–Standard Deviation

*Denotes a significant difference or Oddsratio favoring guideline adherence

NR–Not reported; A–guideline adherent; NA -Non guideline adherent

#### Prescription medications used

Evaluation of the identified studies suggest that patients participating in an adherent physical therapy program used less prescription medication (Table 5). One study reported a 46.2% prescription medication usage in the adherent group, compared to a 57.2% utilization rate in the non-adherent group [38]. Three studies reported utilization rates specifically for opioid medications [15, 17, 38]. One study reported a utilization rate of opioid medications to be 30.3% in the adherent group, compared to 38.1% in the non-adherent group. Fritz et al [15] reported opioid medication usage to be 49.6% in the adherent group, compared to 53.2% in the non-adherent groups. In the study by Childs et al [17] overall opioid medication usage for those adherent to recommended guidelines was 65.2% versus 66.0% in the non-adherent group.

#### Physician office visits

The identified studies [15, 38] suggest utilization of fewer subsequent physician office visits for those participating in an adherent physical therapy program (Table 5). Fritz et al [38] reported additional physician office visits in the year after the episode of physical therapy in the adherent group to be 19.5%, ompared to 25.7% in the non-adherent group, however; this did not demonstrate a statistically significant difference. Another study by Fritz et al [15] reported 64.4% of those participating in an adherent treatment program utilized additional physician office visits in the 18-month period following the index primary care visit, compared to 68.8% in the non-adherent group.

#### Emergency department care

Our review identified one study which reported subsequent emergency department care which supported less utilization of this service for those participating in an adherent physical therapy program (Table 5). Fritz et al [38] reported an overall usage rate of 2.8% in the year after the episode of physical therapy (1.5% for the adherent and 3.2 for the non-adherent group) and the difference was not statistically significant. The other identified studies did not report the rate of emergency department care [15, 17, 37].

#### Advanced imaging

Identified studies support less utilization of advanced imaging for those participating in an adherent physical therapy program (Table 5). In the study by Fritz et al [38] magnetic resonance imaging (MRI) was obtained by 8.3% in the adherent group in the year after the episode of physical therapy, compared to 15.9% in the non-adherent group. A subsequent study published by Fritz et al [15] reported the utilization rate of advanced imaging (MRI or Computed tomography (CT) to be 38.7% in the adherent group in the 18-month period following the index primary care visit, compared to 43.9% in the non-adherent group. Childs et al [17] reported advanced imaging rates to be 17.0% in the adherent group over the 2-year follow up period, compared to 22.7% in the non-adherent group.

#### Surgical procedures

The available evidence reports mixed results for the use of surgical procedures with regards to physical therapy guideline adherence (Table 5). Fritz et al [38] reported a utilization rate of 3.8% in the adherent group in the year after the episode of physical therapy, compared to 3.0% in the non-adherent group. Several years later Fritz et al [15] reported lumbar spine surgery to be 5.1% in the adherent group in the 18-month period following the index primary care visit, compared to 8.1%in the non-adherent group. Finally, Childs et al [17] reported the rate of surgery for those in the adherent group to be 2.6% over the 2-year follow up period, compared to 3.0% in the non-adherent group.

#### Injection procedures

The available evidence suggests reduced use of injection procedures for those participating in an adherent physical therapy program (Table 5). The rate of injection procedures reported by Fritz et al [38] was 9.1% in the adherent group in the year after the episode of physical therapy, compared to 15.9% in the non-adherent group. In 2012, Fritz et al [15] reported a utilization rate for injections to be 12.6% in the adherent group in the 18-month period following the index primary care visit, compared to 17.8% in the non-adherent group. Finally, Childs et al [17] reported injection utilization rates to be 11.7% in the adherent group over the 2-year follow up period, compared to 13.8% in the non-adherent group.

### Healthcare Costs

#### Physical therapy

Based on the available evidence, those participating in adherent physical therapy treatment demonstrated lower costs for physical therapy services (Table 6). A study by Fritz et al [37] reported mean physical therapy charges for patients with LBP were $845.57 for those receiving adherent care, compared to $884.94 for those receive non-adherent care. In another study [38] published in 2008 mean physical therapy charges for those receiving adherent care amounted to $562, compared to $729 for those that received non-adherent care.

**Table 6:** Reports of Healthcare Costs.

	Costs of PT Mean (SD)		Prescription Medication Mean (SD)		Physician Office Visit Mean (SD)		Imaging Procedures Mean (SD)		Surgical/ Injection Procedures Mean (SD)		Inpatient Nonsurgical Procedures Mean (SD)		Inpatient costs Mean (SD)		Total LBP Costs Mean (SD)		Other LBP-Related Costs		Non-LBP Healthcare Costs Mean (SD)		Subsequent Healthcare Costs Mean (SD)	
	A	NA	A	NA	A	NA	A	NA	A	NA	A	NA	A	NA	A	NA	A	NA	A	NA	A	NA
Fritz et al[37] 2007†	$846* ($449)	$885* ($523)	NR	NR	NR	NR	NR	NR	NR	NR	NR	NR	NR	NR	NR	NR	NR	NR	NR	NR	NR	NR
Fritz et al[38] 2008‡	$562* ($269)	$729* ($345)	NR	NR	NR	NR	NR	NR	NR	NR	NR	NR	NR	NR	NR	NR	NR	NR	NR	NR	$1692 ($7683)	$2829 ($21,728)
Fritz et al[15] 2012§	NR	NR	$76 ($10)	$99 ($10)	$296* ($41)	$357* ($10)	$513 ($47)	$701 ($52)	$1,445 ($486)	$1,966 ($229)	$162 ($90)	$143 ($38)	NR	NR	$3,609* ($533)	$4,946* ($277)	$1091* ($89)	$1652* ($53)	$7,255 ($1156)	$7,511 ($402)	NR	NR
Childs et al[17] 2015¶	NR	NR	$886* ($20)	$1,234*($20)	NR	NR	NR	NR	NR	NR	NR	NR	$11,640 ($300)	$11,293 ($249)	$2,427* ($30)	$2,734* ($27)	NR	NR	$9,285 ($90)	$9,157 ($74)	NR	NR

† Costs of PT were related to an episode of care

‡ Costs of PT were related to an episode of care and subsequent healthcare costs were observed in the year after the episode

§ cost measures were observed during the 18-month period following the index primary care visit

¶ cost measures were observed during the 2-year follow-up period

*Denotes a significant difference

NR–Not reported; A–guideline adherent; NA-Non guideline adherent; figures have been rounded to the nearest dollar; SD- Standard Deviation

#### Prescription medications

The identified studies reported a decrease in prescription medication costs for those participating in an adherent physical therapy program (Table 6). One study of individuals consulting a primary care physician for a first episode of care reported that mean prescription medication costs were $76.43 for those receiving adherent physical therapy treatment during the 18-month period following the index primary care visit, compared to $98.85 in those receiving non-adherent interventions [15]. Prescriptions medication costs were substantially higher in another study that evaluated a longer course of care in a military healthcare system [17]. Childs et al [17] reported that those in the adherent group incurred an average $886.27 as prescription medication costs compared with an average $1233.91 for patients in non-adherent group over the 2-year follow-up period.

#### Inpatient costs

Reports of inpatient costs suggest increased costs associated with guideline adherent physical therapy treatment (Table 6). Fritz et al [15] identified inpatient nonsurgical procedures and reported costs for those receiving adherent physical therapy to be an average $162.31 during the 18-month period following the index primary care visit, compared to $142.99 in the non-adherent group. Costs associated with inpatient care (including surgical procedures) reported by Childs et al [17] were an average $11,639.95 in those receiving adherent care over the 2-year follow-up period, compared to $11,292.57 for those in the non-adherent group (Table 6).

#### Total LBP costs

There appears to be cost savings associated with total LBP related expense when patients participate in a guideline adherent physical therapy treatment program (Table 6). Fritz et al [15] reported those patients that received adherent care averaged $1374.30 lower costs when compared to non-adherent care over the 18-month period. Furthermore, Childs et al [17] reported those receiving adherent care had total LBP related costs to be an average $2426.88 over the 2-year period, compared to $2733.57 in the non-adherent group.

#### Non-LBP healthcare costs

Finally, participating in a guideline adherent treatment program appears to have mixed results on cost savings associated with non-LBP related healthcare costs. (Table 6). Fritz et al [15] reported non LBP healthcare costs to be an average $7254.82 in the adherent group over the 18-month period, compared to $7511.44 in the non-adherent group, while Childs et al [17] reported non LBP related healthcare costs in the adherent group to be $9285.09 over the 2-year period, compared to $9157.06 in the non-adherent group.

## Discussion

Physical therapists are common providers of care for t patients with LBP [11]. The available evidence suggested adherence to physical therapy clinical practice guidelines may have an important influence on certain measures of healthcare utilization and costs. With a few exceptions, those patients with LBP that were treated via an adherent guideline program demonstrated an association with decreased healthcare utilization and an overall healthcare savings [37, 38]. Specifically, it appears those participating in an adherent physical therapy treatment program results in lower overall healthcare utilization including fewer PT visits, shorter duration of care, fewer prescription medications, fewer visits to the physicians or emergency department and less use of advanced imaging and injection procedures. Also, decreased costs have been associated with PT services, prescription medications, additional physician office visits, advanced imaging, surgical procedures as well as total LBP related costs for those patients with LBP treated by guideline adherent physical therapy interventions. Those studies reporting total LBP costs demonstrated a cost savings of between $300 and $1300.00 US dollars which would entail significant healthcare costs savings if administered consistently in the population with LBP [15, 17]. While one study reported elevated inpatient costs associated with guideline adherence the difference was minimal and when considering variability in the measure there appears to be no difference.

### Definitions of Guideline Adherence

It has been suggested that CPG decrease utilization of ineffective treatments, adopt practices that are evidence-based, and improve patient outcomes [10]. They are intended to guide clinical decision making but not necessarily dictate specific approaches to different types of conditions. This creates a challenge for researchers as there may be a multitude of ways to identify guideline adherent interventions, which can ultimately make it difficult to track. However, when evaluating CPG there are some common threads. Specifically, treatments should generally focus on active treatment approaches (e.g therapeutic exercise) and limit passive treatments. Fritz et al [37] reviewed current practice guidelines and developed a method of using claims data to identify treatment patterns which were compliant. Specifically, they divided episodes of care into two phases. Phase I reflected treatment for the first two weeks of care and phase II included treatment from day 14 to the end of the episode of care. Guideline recommendations generally suggest that treatment approaches may vary during these different phases and still be guideline adherent. It was established that overall adherence required adherence in both phase I and phase II. Adherence was defined based on Current Procedural Terminology (CPT) codes billed for each visit and were classified as active, passive, or allowable. Active codes reflected active treatment procedures while passive codes suggested the patient played a passive role in the treatment. Allowable codes were those codes which could not be classified as active or passive and included evaluation, testing or equipment codes. Fritz et al [37] identified the number of active and passive codes at each visit and the percentage of active to passive codes was calculated [number of active codes/ (number of active codes + number of passive codes) X 100%. Adherent care was defined as having at least 75% active to passive codes for both phases and each visit included at least 1 active code. Each of the identified studies in this review used the same methodology in reporting adherent vs. non-adherent care.

### Healthcare Utilization

The majority of studies show a small difference in the number of PT visits between those participating in an adherent and non-adherent treatment program. For three studies [15, 37, 38] the difference ranged from 1 to 2 fewer visits for those participating in an adherent care program. However, the study by Childs et al [17] revealed a large difference of almost 9 visits. Little difference existed for prescription medication use as did differences for additional physician office visits. Also, while the use of subsequent emergency department care was low (approximately 3% in the sample), there was little difference between those participating in an adherent versus non-adherent program. However, significant differences in advanced imaging was reported between those participating in an adherent and non-adherent treatment program. There seems to be conflicting evidence with regards to surgical procedures. While two studies [15, 38] demonstrated fewer surgical cases, another study [17] demonstrated an increase in surgical cases for those participating in an adherent physical therapy program. One might speculate that surgical cases are more likely to be appropriately identified among those being treated with adherent care as credentialing of the provider is seemingly linked to providing adherent care [9].

The Study by Childs et al [17] did show significant adjusted odds ratios for specific utilization outcomes. When evaluating adherent vs. non-adherent treatment, advanced imaging was 0.72 (0.69,0.76), spinal injection, 0.82 (0.77, 0.87), lumbar surgery 0.85, (0.75,0.96) and opioid use, 0.97 (0.93, 1.01). Of interest in this study is it seems that both Content (Adherent vs. Non-Adherent and timing (early vs. delayed) have a significant impact on utilization as those that were treated in physical therapy within 1- days with guideline adherent interventions demonstrated less healthcare utilization and cost.

### Healthcare Costs

The costs of physical therapy services were generally lower for those participating in adherent care. This was also consistent with other interventions such as use of medications and consistent with the utilization reports. Reports of inpatient and non-LBP related costs varied. Of particular interest, are those participating in adherent care, who demonstrated increased inpatient costs. Fritz et al [15] and Childs et al [17] both reported increased costs for inpatient care in those participating in adherent physical therapy treatment. Although the difference does not seem to be substantial, it is inconsistent with other LBP associated costs and may simply represent early identification of more serious cases needing such care. While, there may be some cost saving for LBP related healthcare costs it does not appear to be substantial. Also, one study [15] reported a savings with regards to non-LBP related healthcare and another study [17] reported increased non-LBP costs associated with adherence physical therapy program.

### Influence of Other Guideline Adherence Studies

This concept has been true for other clinical treatment guideline recommendations. For example, it has been recommended that expensive diagnostic tests be deferred in the absence of red flag findings in those with LBP. In an attempt to evaluate the influence of these recommendations Graves et al [39] evaluated 1,770 workers reporting LBP and found that 19% were classified as non-adherent to recommended guidelines for MRI. They also found that utilization of outpatient rehabilitation services were over 50% higher for those individuals that received non-compliant imaging. Ultimately, the authors reported that non-adherence to guidelines for use of MRI was associated with a higher rate of treatment, which included injections, surgery and higher costs for other medical services. Furthermore, those individuals that did receive advance imaging early had a higher likelihood of disability and duration of LBP [40]. It appears there are multiple opportunities to influence healthcare savings and patient outcomes by being aware of recommended guidelines for those with LBP. Current evidence suggests many similarities in clinical practice guidelines which include gradual return to activity and avoidance of bed rest, as well as identification of psychosocial risk factors for chronicity [12]. This suggests that guidelines for creating a multidisciplinary LBP approach may have broader implications [41].

### Limited Approaches to Defining Guideline Adherence

A limited number of studies have used claims data to evaluate guideline adherence. Initially proposed by Fritz et al in 2007 [37], this methodology for defining adherent versus non-adherence care may have limitations. Also, all papers identified in this review were based on the same methodology for defining guidelines adherence. This provides a limited scope in our understanding of how guideline adherence may be defined. While this approach does capture general adherence criteria, there is a limited scope of understanding when all of the current studies evaluating guideline adherence have been through a single lens. While adherence was based on the best available evidence, the data provides little guidance to the practicing physical therapist regarding specific interventions. Interventions billed under stated active codes can vary significantly. Therefore, it is not clear what mechanisms may have had the greatest influence on healthcare utilization and costs. Also, those patients that are higher functioning are more likely to participate in an active treatment program [42, 43]. These patients are naturally going to demonstrate decreased episodes of care as well as a decrease in overall cost. Patients with a more severe condition may not be able to fully participate with in interventions aligned with active codes and thus a higher percentage of these patients may have more passive codes billed. Therefore, the reasoning for decreased cost of care for those billed for more active units may be due to a better potential for a positive outcome to begin with and not specifically because of the intervention.

### Reasons for Non-Adherence to Guidelines

There are a variety of reasons why clinicians do not comply with recommended guidelines. One survey identified varied understanding of CPGs when making decisions regarding CPGs [44]. Specifically, there were four primary reasons which included: understanding of CPG); compatibility of guidelines with current practices; level of perceived relevance to the clinician, and level of agreement with CPGs [45]. As an example, one study identified a biomedical orientation to clinical decision making which were predictive of clinical judgments which was associated with intolerance of uncertainty [46]. A behavioral approach predicted treatment approaches which recommended return to work or increased levels of activity [46]. Adherence to recommended CPG improved if the there was an active approach to training [47]. Improved clinical outcomes and reduced cost was associated with adherence to physical therapy clinical practice guidelines (CPGs) for active care [37].

### Limitations

The articles identified in this systematic review were retrospective analyses of existing data sets and can only infer associations and not causation. Also, other types of guideline adherence were not considered in this review and therefore other interventions should be evaluated independently. While there are alternate explanations for differences, it is impossible to determine if clinicians included were more experienced with the concept of guideline adherence and therefore better able to established therapeutic alliances with their patients enhancing recovery and necessitating decreased healthcare utilization.

This review reports on studies that implemented a single manner by which to identify adherent vs. non-adherent care. Namely, the use of active vs. passive modalities. While this is due to limitation of the data sets, it could be argued that while PT guidelines recommend active treatment, the identified studies did not directly measure compliance and simply used an indirect measure to infer compliance with guideline adherence. Finally, there is some difficulty in comparing results to other reviews as this is the first to evaluate guideline adherence utilization the same methodology. To date, no other reviews have evaluated utilization of active vs. passive physical therapy interventions on healthcare costs and utilization using claims data.

## Conclusion

Adherence to established clinical practice guidelines may assist with decreasing healthcare utilization and costs. However, it can be challenging to evaluate specific practice patterns on a large scale. Proposed evaluation of claims data may assist in determining the most cost effective approach to treatment. While this review indicated mixed results regarding some cost measures, patients participating in an adherent physical therapy treatment program were associated with reduced healthcare utilization and lower costs. However, there are limitations to this approach and future research should evaluate outcomes in a randomized clinical trial to establish the magnitude of effectiveness on healthcare utilization and costs. One challenge resides in the heterogeneous presentation of patients seeking care. Particularly as those with more serious presentations (e.g. hard neurological signs or cauda equina syndrome) are likely to have a greater cost of inpatient care irrespective of adherence.

## Supporting Information

S1 Table Modified Downs & Black Scale. (DOCX)
