# Supplementary material for: The Influence of Physical Therapy Guideline Adherence on Healthcare Utilization and Costs among Patients with Low Back Pain: A Systematic Review of the Literature
Source: PLoS One. 2016 Jun 10;11(6):e0156799. doi: 10.1371/journal.pone.0156799 (PMC4902217; doi:10.1371/journal.pone.0156799)
Supplement: S1 Table — (DOCX) [file pone.0156799.s001.docx]

# Supplement Table 1 – Modified Downs & Black Scale

| **Number** | **Criteria** | **Scoring Criteria** | **Score** |
| --- | --- | --- | --- |
| 1 | Is the hypothesis/aim/objective of the study clearly described? | A point was given if the hypothesis aim or objective of the study was implicitly or explicitly indicated anywhere in the article. | 0 = No 1 = Yes |
| 2 | Are the main outcomes to be measured clearly described in the "Introduction" or "Methods" section? | A point was given if the main outcomes to be measured were clearly described in the "Introduction" or "Methods" section. | 0 = No 1 = Yes |
| 3 | Are the characteristics of the patients included in the study clearly described? | A point was given if the inclusion or exclusion criteria, or both, were indicated. | 0 = No 1 = Yes |
| 4 | Are the interventions of interest clearly described? | A point was given if the criteria for guideline adherence were described in detail | 0 = No 1 = Yes |
| 5 | Are the distributions of principal confounders for each group of participants to be compared clearly described? | Two points were awarded if a study reported any possible confounders (e.g., sex ratios, age, comorbidities, and severity of injury) that might account for differences between groups clearly in table format. One point was awarded if the study indicated that groups were matched for any such demographical variables or if potential confounders were mentioned in the text of the article but not clearly listed in table format. No points were awarded if the study did not report any confounders. | 0 = No 1 = Partially 2 = Yes |
| 6 | Are the main findings of the study clearly described? | A point was awarded if quantitative data were reported for all of the main outcome measures indicated in the "Introduction" or "Methods" section. | 0 = No 1 = Yes |
| 7 | Does the study provide estimates of the random variability in the data for the main outcomes? | A point was awarded if the interquartile range (for non-normally distributed data), standard error, standard deviation, or confidence intervals (for normally distributed data) were reported. If the distribution of the data was not described, we assumed that the estimates used were appropriate, and we answered "yes" (1 point). | 0 = No 1 = Yes |
| 8 | Have all of the important adverse events that may be a consequence of the intervention been reported? | A point was awarded if any adverse events, unwanted side effects, or lack thereof were explicitly indicated from either adherence or failure to adhere to recommended guidelines. A point was not awarded if the study made no mention of the presence or absence of adverse events. | 0 = No 1 = Yes |
| 9 | Have the characteristics of patients lost to follow-up been describe? | The authors of this tool indicated that this question should be answered “yes” when clear reasons for loss to follow-up were described. For the purposes of this review, a point was awarded if a study explicitly reported the number and reason for patients lost to follow-up. | 0 = No 1 = Yes |
| 10 | Have actual probability values been reported (e.g. 0.035 rather than < 0.05) for the main outcomes except where the probability value is less than 0.001? | A point was awarded if the exact P value was provided for both statistically significant and non-significant results for at least the main outcome measures. A point was not awarded if a study simply indicated that the results for the main outcome measures were not significant without providing the exact P value. | 0 = No 1 = Yes |
|  | **EXTERNAL VALIDITY** |  |  |
| 11 | Were the subjects asked to participate in the study representative of the entire population from which they were recruited? | A point was awarded if the study identified the source population for patients and described how the patients were selected. Patients were determined to be representative if they comprised the entire source population, an unselected sample of consecutive patients, or a random sample (only feasible where a list of all members of the relevant population exists). Where a study did not report the proportion of the source population from which the patients are derived, the question was answered as unable to determine. | 1 = Yes 0 = No 0 = Unable to determine |
| 12 | Were those subjects who were prepared to participate representative of the entire population from which they were recruited? | The proportion of patients included in the study were representative of the population. Those asked who agreed to participate or responded should be stated. Validation that the sample was representative would include demonstrating that the distribution of the main confounding factors was the same in the study sample and the source population. No point was awarded if the proportion of those asked who agreed to participate or responded was not stated. | 1 = Yes 0 = No 0 = Unable to determine |
| 13 | Were the staff, places and facilities where the patients were treated, representative of the treatment the majority of patients receive? | A point was awarded unless the study specifically stated that patients were treated by a therapist who received specialized training relative to guideline recommendations. | 1 = Yes 0 = No 0 = Unable to determine |
|  | **INTERNAL VALIDITY - BIAS** |  |  |
| 14 | Was an attempt made to blind study subjects to the intervention they have received? | A point was awarded if the patients were not aware of, or would have no way of knowing (as in the case of retrospective studies), which intervention they received. The study was not awarded a point if it was prospective and failed to mention whether the patients had knowledge of whether they were assigned to the guideline adherence group. | 1 = Yes 0 = No 0 = Unable to determine |
| 15 | Was an attempt made to blind those measuring the main outcomes of the intervention? | A point was awarded if the study specifically stated that those assessing the outcome measures were unaware of (or would have no way of knowing) whether the patients were in the guideline adherence group. | 1 = Yes 0 = No 0 = Unable to determine |
| 16 | If any of the results of the study were based on "data dredging", was this made clear? | A point was awarded if no retrospective unplanned (at the outset of the study) subgroup analyses were reported. | 1 = Yes 0 = No 0 = Unable to determine |
| 17 | In trials and cohort studies, do the analyses adjust for different lengths of follow-up of patients, or in case-control studies, is the time period between the intervention and outcome the same for cases and controls? | For the purposes of this review, this question was omitted due to reasons previously stated. | N/A |
| 18 | Were the statistical tests used to assess the main outcomes appropriate? | If the distribution of the data (normal or not) was not described, it was assumed that the estimates used were appropriate, and a point was awarded. No point was awarded for studies that reported qualitative or quantitative data without any form of statistical comparisons or if the statistical tests reported were not appropriate. | 1 = Yes 0 = No 0 = Unable to determine |
| 19 | Was compliance with the intervention/s reliable? | If the authors in prospective studies reported non-adherence to physical therapy intervention or adherence could not be determined, the study was not awarded a point. In retrospective studies, data were collected only for those patients who completed their episode of care (adherence to physical therapy assumed), and a point was awarded. For studies where the effect of any non-adherence was likely to bias any association to the null, the study was not awarded point. | 1 = Yes 0 = No 0 = Unable to determine |
| 20 | Were the main outcomes measures used accurate (valid and reliable)? | A point was awarded if the primary outcome measures were thought to be valid and reliable (e.g., number of physical therapy visits per chart report), regardless of whether reliability or validity was reported. A point was not awarded if at least one of the primary outcome measures in the study was not valid or reliable or if this information was not reported or could not be determined (i.e., a questionnaire without reported validity or reliability). | 1 = Yes 0 = No 0 = Unable to determine |
|  | **INTERNAL VALIDITY - CONFOUNDING (SELECTION BIAS)** |  |  |
| 21 | Were the patients in different intervention groups (trials and cohort studies) or were the cases and controls (case-control studies) recruited from the same population? | A point was awarded when participants from both adherence and non-adherence groups were recruited from the same population. Otherwise, a point was not awarded (e.g., a point was not awarded when all participants from the adherence group received care at clinic A and all participants in the non-adherence group received care at clinic B, because they could have represented 2 distinct populations). | 1 = Yes 0 = No 0 = Unable to determine |
| 22 | Were study subjects in different intervention groups (trials and cohort studies) or were the cases and controls (case-control studies) recruited over the same period of time? | A point was awarded when the study provided a specific time line for patient recruitment (prospective studies) or when data were collected between reported dates of patient care (retrospective studies). | 1 = Yes 0 = No 0 = Unable to determine |
| 23 | Were study subjects randomized to intervention groups? | A point for random allocation was awarded if random allocation of patients was stated in the “Method” section of the article. The precise method of randomization need not be specified. Quasi-randomization allocation procedures, such as allocation by bed availability, did not satisfy this criterion. For crossover study designs, a point was awarded when participants were randomly allocated in the order in which treatments were received. | 1 = Yes 0 = No 0 = Unable to determine |
| 24 | Was the randomized intervention assignment concealed from both patients and healthcare staff until recruitment was complete and irrevocable? | The study did not receive a point unless the participants were randomly allocated and the methods for ensuring random allocation were specified. | 1 = Yes 0 = No 0 = Unable to determine |
| 25 | Was there adequate adjustment for confounding in the analyses from which the main findings were drawn? | A point was awarded unless the effect of the main confounders was not investigated or confounding was demonstrated, but no adjustment was made in the final analyses. | 1 = Yes 0 = No 0 = Unable to determine |
| 26 | Were losses of patients to follow-up taken into account? | A point was awarded as long as the number of dropouts lost to follow-up accounted for less than 10% of the initial number of total participants or a maximum of 5% from each group. The question was answered with “unable to determine” if the number of patients lost to follow-up were not reported, could not be deduced from the outcome data (i.e., initial and final sample sizes not indicated) or the study methodology would not infer such information. | 1 = Yes 0 = No 0 = Unable to determine |
|  | **POWER** |  |  |
| 27 | Did the study have sufficient power to detect a clinically important effect where the probability value for a difference being due to chance is less than 5%? | For the purposes of this evidence-based review, this question was omitted. | N/A |
|  |  | **Overall Max Possible Score** | 26 |
